# Role of the *gerA *operon in L-alanine germination of *Bacillus licheniformis *spores

**DOI:** 10.1186/1471-2180-12-34

**Published:** 2012-03-15

**Authors:** Irene S Løvdal, Cecilie From, Elisabeth H Madslien, Kristin Cecilia S Romundset, Elin Klufterud, Jan Thomas Rosnes, Per Einar Granum

**Affiliations:** 1 Nofima AS, Department of Process Technology, Måltidets hus, Richard Johnsens gate 4, P. Box 8034, N-4068 Stavanger, Norway; 2 Departement of Food Safety and Infection Biology, Section for Food Safety, Norwegian School of Veterinary Science, Ullevålsveien 72, P. Box 8146 Dep., N-0033 Oslo, Norway; 3 Forsvarets Forskningsinstiutt FFI, Norwegian Defence Research Establishment, P. O. Box 25, N-2027 Kjeller, Norway; 4 Current address; University of Stavanger, Faculty of Arts and Education, Department of Early Childhood Education, N-4036 Stavanger, Norway; 5 Current address; Mills DA, Sofienberggata 19, P. Box 4644, N-0506 Oslo, Norway

## Abstract

**Background:**

The genome of *Bacillus licheniformis *DSM 13 harbours three neighbouring open reading frames showing protein sequence similarities to the proteins encoded from the *Bacillus subtilis *subsp. *subtilis *168 *gerA *operon, GerAA, GerAB and GerAC. In *B. subtilis*, these proteins are assumed to form a germinant receptor involved in spore germination induced by the amino acid L-alanine.

**Results:**

In this study we show that disruption of the *gerAA *gene in *B. licheniformis *MW3 hamper L-alanine and casein hydrolysate-triggered spore germination, measured by absorbance at 600 nm and confirmed by phase contrast microscopy. This ability was restored by complementation with a plasmid-borne copy of the *gerA *locus. Addition of D-alanine in the casein hydrolysate germination assay abolished germination of both *B. licheniformis *MW3 and the complementation mutant. Germination of both *B. licheniformis *MW3 and the *gerA *disruption mutant was induced by the non-nutrient germinant Ca^2+^-Dipicolinic acid.

**Conclusions:**

These results demonstrate that the *B. licheniformis *MW3 *gerA *locus is involved in germination induced by L-alanine and potentially other components present in casein hydrolysate.

## Background

Germination of dormant *Bacillus *spores and subsequent outgrowth can be induced by various nutrients (amino acids, purine nucleosides, sugars, ions and combinations of these) recognised by receptor proteins encoded by the *gerA *family operons [1-3] and located in the inner membrane of the spore [4-7]. One or several germination receptor operons have been detected in the genomes of almost all spore formers, and supported by studies of different mutants it has been concluded that spores respond to germinants via receptors diverged from common ancestor(s) ([6] and references therein). Studies of receptor/germinant interactions have so far mainly been focusing on species belonging to *Bacillus cereus, Bacillus subtilis, Bacillus megaterium *and *Bacillus anthracis *[3,8-16]. *Bacillus licheniformis*, another Gram-positive, spore forming soil bacterium closely related to *B. subtilis *[17], has on the other hand gained much less attention. *B. licheniformis *is a frequent contaminant of foods, and is a common spoilage organism of dairy products [18-20], bread [21,22], packaged meats [23] and canned goods [24]. It has previously been considered non-pathogenic, and has been widely used in the industry for production of enzymes, antibiotics and biochemicals [25-27]. However, *B. licheniformis*-associated bovine abortion [28,29], implant infection [30], corneal ulcer [31], bacteraemia sepsis [32] and food poisoning [33,34] raise the question of its pathogenic potential. Some strains of *B. licheniformis *associated with human disease are capable of producing lichenysin A, a surfactin-like toxin [34,35]. Due to its association with food-borne illness and spoilage, and its ability to undergo sporulation, [17,36-38], extended knowledge about the germination apparatus of *B. licheniformis *is of general interest. To ensure microbiological safe food production of durable foods produced by relatively mild heat treatment, there is an obvious need for more information on spore forming bacteria.

Based on existing literature, *B. subtilis *could be considered as the model organism for germinant receptor studies. It was through early studies of germination defective mutants, that the theory of a L-alanine-induced germinant receptor was proposed [8]. Later studies identified the *gerA *locus as a tricistronic operon weakly expressed during sporulation, and that the polypeptide products of *gerA *probably formed a membrane associated complex [39-41]. The products of each of the three genes of *gerA *were later named GerAA, GerAB and GerAC, and were demonstrated to be simultaneously required for the spore to respond to L-alanine as sole germinant [2]. Genome sequence analysis and germination experiments of different mutants further identified four other tricistronic *gerA *homologs for *B. subtilis; gerB, gerK, yndDEF *and *yfkQRT *[10]. Receptors encoded by two of these operons, *gerB *and *gerK*, are confirmed functional when acting cooperatively with each other or with gerA [10,15].

Homologous genes of germinant receptors belonging to the *gerA *family have been found in most spore formers, although the exact number, organisation and corresponding response germinant may vary for different species and even strains [3,42,43]. *B. licheniformis *ATCC 14580 is also predicted to possess potential germinant receptor proteins belonging to both the GerA and the GerK clades [44]. The GerAA, GerAB and GerAC protein sequences of *B. licheniformis *ATCC14580 are closely related to the protein sequences of the corresponding germinant receptor subunits of *Bacillus subtilis *subsp. *subtilis *168. These are in *B. subtilis *encoded by the *gerA *operon, *gerAA, gerAB *and *gerAC*. Since *B. subtilis gerA *germination is triggered by L-alanine [2,15], it is plausible that the *B. licheniformis gerA *operon also is involved in L-alanine germination. It has earlier been documented that spores of *B. licheniformis *from different strains actually respond to L-alanine as germinant [45-47], but to our knowledge, there are no functional studies of receptor/germinant interactions of strains belonging to *B. licheniformis*.

Mutational studies of *B. licheniformis*, including the fully sequenced *B. licheniformis *ATCC 14580/DSM 13 strain [48,49], have long been a challenge, most likely due to their possession of a restriction apparatus destroying foreign DNA [48-50]. The construction of a more easily transformable mutant, *B. licheniformis *MW3, has largely overcome this challenge [50].

In order to facilitate the understanding of germinant/receptor interactions in *B. licheniformis*, we have constructed disruption and complementation mutants of the *gerAA *locus in *B. licheniformis *MW3. Spores of these mutants have been studied in germination assays with L-alanine, casein hydrolysate and the non-nutrient germinant Ca^2+^Dipicolinic acid (Ca^2+^DPA). These studies reveal that gerA is a main germinant receptor complex of *B. licheniformis *recognising amino acid(s), and supports the view that L-alanine is an important nutrient-germinant for this species.

## Results and Discussion

### Construction of the disruption and complementation mutants

To elucidate the role of the hypothetical GerA proteins during spore germination, a disruption mutant of the *gerAA *locus in *B. licheniformis *MW3 was constructed. *B. licheniformis *MW3 was used as target strain due to its superior transformability compared to its fully sequenced parent strain DSM 13 [50]. The *gerAA *mutant, NVH-1307, was constructed so that a part of the *gerAA *gene was substituted with a spectinomycin resistance cassette. This will cause the mutant to acquire spectinomycin resistance, and in addition, affect a potential phenotype related to the disrupted gene. If the target gene is part of an operon, which is the case of *gerAA*, downstream transcripted genes will also be affected, and the receptor non functional. Sequence analysis showed that in addition to harbouring the spectinomycin cassette in the *gerAA *locus, NVH-1307 also harboured two additional mutations (one base substitution and one base deletion) in the *gerAA *locus. These mutations were most likely acquired during PCR amplification of the fragments used to construct the disruption vector (pMAD_Sp^R^Δ*gerAA*). These mutations were "accepted" (not corrected) due to their location in the gene targeted for disruption. However, in construction of the plasmid used for *gerAA *complementation, a polymerase with a higher expected fidelity was applied to limit the risk of such mutations. Sequence analysis of the complementation plasmid pHT315_MW3*gerA *revealed no mutations in the amplified *gerA *operon when compared to the sequence of Veith et al.[48].

Genetic modification studies have shown that the germination rates could be significantly increased when specific germinant receptors are over-expressed in *B. subtilis *[51]. Thus, expression of germinant receptors is apparently not optimised for maximal spore germination, forwarded as a possible evolutionary strategy to prevent premature germination at nutrient conditions inadequate for sustained vegetative growth [3]. Very high levels of receptor expression could on the other hand have a negative effect on the sporulation process [51]. In such cells, the forespore lyses during the process of sporulation, perhaps as a result of premature forespore germination [51]. An appropriate evolutionary adaptation of germinant receptor expression/regulation is thus crucial to allow the cyclic transition between sporulation and germination upon environmental changes. In the construction of the complementation mutants in our study, certain precautions were therefore taken to avoid extensive over-expression of the complemented germinant receptor genes. By including some of the flanking regions of the *gerAA, gerAB *and *gerAC *fragment in the complementation plasmid, we wanted to maintain the native regulatory elements of this locus. In addition, a shuttle-vector with an expected low or moderate copy number was sought as a basis for the complementation plasmid. To our knowledge, there is no shuttle-vector available for *B. licheniformis *where the copy number is demonstrated to be low or moderate. However, Arantes and Lereclus [52] have constructed the pHT315 *E. coli/B. thuringiensis *shuttle-vector, with a copy number of ~ 15 per equivalent *B. thuringiensis *chromosome. This vector has successfully been used in germinant receptor complementation studies in *B. megaterium *[53], and was thus considered as a reasonable choice for *B. licheniformis*. Despite that this vector has shown to be stably maintained in *B. thuringiensis *and *B. megaterium *without a selective pressure [52,54], the antibiotic erythromycin had to be included to ensure persistence of the complementation plasmid during sporulation of the *B. licheniformis *complementation mutant NVH-1311. This could be due to a different segregation stability of the vector in *B. licheniformis*. Another possibility is that there is a potential elevated risk of plasmid curing due to sporulation at a high temperature. Sporulation of *B. licheniformis *MW3, NVH-1307 and NVH-1311 were performed at 50 °C since a pilot study showed that sporulation at this temperature was faster, yielded more stable spores (less spontaneous germination) and a higher percentage of phase bright spores (results not shown).

### Disruption of *gerAA *abolish L-alanine and casein hydrolysate induced germination

**Figure 1:**
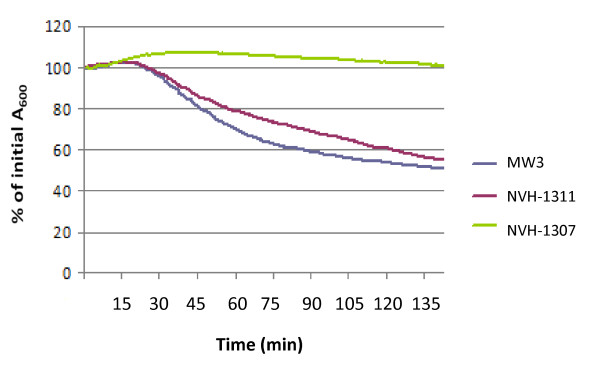
**Germination of *B. licheniformis *with L-alanine**. Germination is followed as a change in initial absorbance at 600 nm (A_600_) of phase bright spores in K-phosphate buffer pH 7.2 at 30 °C after addition of 100 mM L-alanine. Complete germination (>99% phase dark spores as observed by phase contrast microscopy) was observed at ~40% of initial A_600_. The results shown are representative of experiments performed in duplicate on two individual spore batches repeated at least twice.

**Figure 2:**
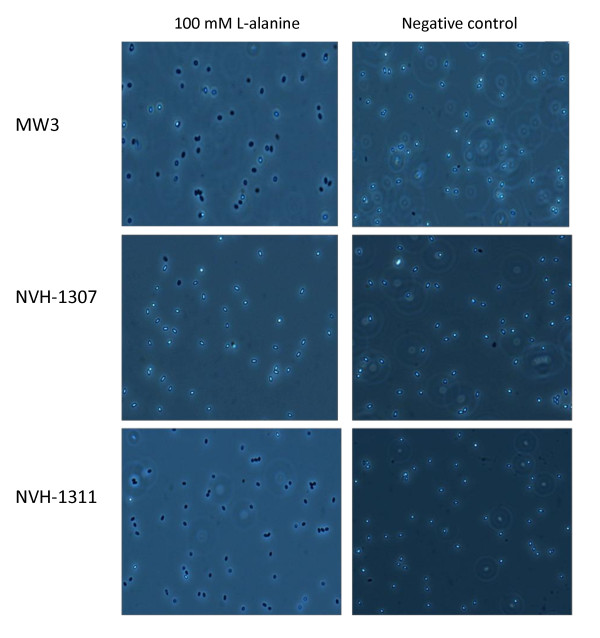
**Phase contrast images of *B. licheniformis *spores following L-alanine germination**. Phase contrast images (100 x) showing *B. licheniformis *spores after 3 hours germination at 30 °C with 100 mM L-alanine or negative control (MQ) in K-phosphatebuffer pH 7.2. The displayed images are representative of experiments performed in duplicate on two individual spore batches repeated at least twice.

Decrease in absorbance at ~ 600 nm (A_600_) is used as a convenient method to monitor and compare germination of different spore populations [55,56]. A fall in absorbance reflects a change in the refractive index (light scattering) of the multiple individual spores in a suspension, associated with germination events such as the excretion of spore's depot of Ca^2+^-DPA, followed by water influx, cortex degradation and core swelling [51,56-59]. Figure 1 shows a representative experiment where different strains of heat activated (65 °C 20 min) spores (in Phosphate buffer) are supplemented with the germinant L-alanine. At these conditions, a clear change in absorbance was observed for spores of wild type (MW3) and wild type complementation mutant (NVH-1311) supplemented with L-alanine. Less than a 5%/h decrease in absorbance was observed for spores of the disruption mutant (NVH-1307). Phase-contrast images captured at the end of the germination assay (Figure 2), a technique where germinated and dormant spores appear dark and white/bright respectively [56,59,60], supports the absorbance measurements showing that spores of the wild type (MW3) and wild type complementation mutant (NVH-1311) germinate with L-alanine. No apparent increase in number of phase dark spores was observed for spores of the deletion mutant (NVH-1307) supplemented with L-alanine, or the negative controls. Together with the absorbance measurements, this shows that the introduced disruption of the *gerAA *gene abolishes the ability of *B. licheniformis *MW3 to use L-alanine as a germinant. The fact that the NVH-1311 complementation mutant showed a similar L-alanine triggered germination phenotype as the wild type spores, supports the hypothesis that an undisrupted copy of the *gerAA, gerAB *and *gerAC *genes, with flanking elements, are required for normal germination of *B. licheniformis *MW3 at these conditions. These findings were also supported by experiments performed with an alternative germination buffer; 50 mM Tris HCl pH 7.4 10 mM KCl (E. Klufterud, C. From; unpublished results).

**Figure 3:**
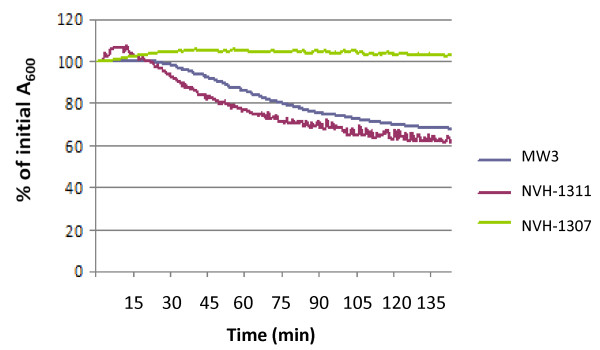
**Germination of *B. licheniformis *with casein hydrolysate**. Germination is followed as a change in initial absorbance at 600 nm (A_600_) of phase bright spores in Tris HCl buffer pH 7.4 at 30 °C after addition of 1% (w/v) casein hydrolysate. Complete germination (>99% phase dark spores as observed by phase contrast microscopy) was observed at ~40% of initial A_600_. The results shown are representative of experiments performed in duplicate on two individual spore batches repeated at least twice.

An earlier study where germination in seven strains of *B. licheniformis *was investigated, showed that out of 24 amino acids tested, only L-alanine, L-cysteine and L-valine markedly stimulated germination [46]. In general, a greater germination response with L-alanine than with L-cysteine and L-valine was observed [46]. To assay the germination response of MW3, NVH-1307 and NVH-1311 to several amino acids, casein hydrolysate was used. Casein hydrolysate consists of a mixture of amino acids made from acid hydrolyzation of the milk protein casein and has been used as a germinant for *Clostridium bifermentans *and *B. cereus *in earlier studies [61-63]. In our study, casein hydrolysate proved to be a potent germinant for *B. licheniformis*, giving a rapid germination response (~70% phase dark spores as visualised by phase contrast microscopy) both for the wild type MW3 and the complementation mutant NVH-1311. The mutant NVH-1307, which most likely lacks a gerA receptor, did not show any germination response in casein hydrolysate (Figure 3).

D-alanine is a well-known inhibitor of L-alanine germination of *B. subtilis *and *B. licheniformis *[64,46,15,66]. D-alanine has also been shown to reduce L-valine induced germination of *B. subtilis *[15,66], but we are not aware of studies reporting the effect of D-alanine on L-valine induced germination of *B. licheniformis*. In order to abolish germination by L-alanine present in the casein hydrolysate, we added D-alanine in some of the above experiments. In these experiments, the germination response of both MW3 and NVH-1311 was hardly measurable (results not shown), indicating that L-alanine through its triggering of the gerA receptor is an important germinant of *B. licheniformis*. The contribution to germination of the remaining amino acids in the casein hydrolysate when D-alanine was present, appear to be minimal. Although one can not rule out that D-alanine also inhibits the effect of other amino acids present in casein hydrolysate (e.g. L-valine), all the findings support the view that gerA and L-alanine constitute one of the main germination pathways of *B. licheniformis*.

### Germination of *B. licheniformis *with Ca^2+^-DPA

In order to by-pass the spore germination receptor apparatus, experiments using exogenous Ca^2+^-DPA to trigger germination of spores of *B. licheniformis *MW3 and the mutant strain NVH-1307 were performed. In *B. subtilis *spores, Ca^2+^-DPA induced germination is believed to act through activation of the cortex lytic enzyme CwlJ, without any requirement of functional germinant receptors [10,67]. Bioinformatic analysis of complete genomes of different spore formers has shown that also *B. licheniformis *contains a *B. subtilis *homologous *cwlJ *gene [43]. If the germination apparatus of *B. licheniformis *spores is similar to that of its close relative *B. subtilis*, the wild type and disruption mutant of *B. licheniformis *should exhibit a similar germination response as *B. subtilis *to exogenous Ca^2+^-DPA. The DPA concentration needed to trigger germination in *B. subtilis *is ~ 20 - 60 mM, supplemented together with equal (or excess) amounts of Ca^2+ ^(allowing formation of a 1:1 chelate of calcium and dipicolinic acid) [10]. Also spores of *B. cereus *and *B. megaterium *germinate when exposed to Ca^2+^-DPA [68,69]. For *B. cereus *it has been shown that a final level of 60 mM Ca^2+^-DPA is sufficient to ensure germination [69]. In our experiments (Table 3), *B. licheniformis *spores of MW3, the mutant NVH-1307 and *B. subtilis *spores of strain B252 (used as a positive control) germinated effectively after 3 hours exposure in room temperature at a final concentration of 80 mM DPA and 100 mM CaCl_2_. Further, at 45 mM DPA 50 mM CaCl_2 _spores of *B. cereus *ATCC 14579 germinated effectively whilst spores of *B. subtilis *strain B252 showed a moderate germination response. *B. licheniformis *MW3 and NVH-1307 exhibited a weak germination response even after a prolonged exposure of ~21 h at these concentrations. At 20 mM DPA 30 mM CaCl_2 _*B. cereus *ATCC 14579 germinated moderately whilst spores of MW3, NVH-1307 and *B. subtilis *B252 did not germinate (Table 3). Earlier Ca^2+^-DPA germination studies with other *B. licheniformis *strains in our collection have yielded similar results with less effective Ca^2+^-DPA induced germination compared to *B. cereus *ATCC 14579 and spores of *B. pumilus *(results not shown). Reasons for a reduced sensitivity to Ca^2+^-DPA as a non-nutrient germinant in *B. licheniformis *MW3 spores compared to spores of some other spore forming bacteria is unknown. It might be that the relationship between Ca^2+ ^and DPA or the concentration of the chelate is not ideal for *B. licheniformis *germination. Another possibility is that a so far uncharacterised non-nutrient inducing germinant or a mixture of DPA with other ions than Ca^2+ ^is needed for effective CwlJ mediated germination of *B. licheniformis*. It has been shown in earlier studies that for instance strains of *B. megaterium *also germinate in mixtures with other ions than Ca^2+ ^[70]. More information on CwlJ and other enzyme interactions with Ca^2+^-DPA is needed to get a clear view on which mechanisms form the basis for the different effects of Ca^2+^-DPA germination in *B. licheniformis, B. cereus *and *B. subtilis*. Further characterisation of Ca^2+^-DPA dependent germination of *B. licheniformis *is currently carried out by our group.

## Conclusions

As demonstrated by genetic mutation and complementation analysis, this study reveals that the *gerAA *gene in *B. licheniformis *MW3 has a fundamental role in germination triggered by L-alanine and casein hydrolysate. We also show that D-alanine is an important inhibitor in *B. licheniformis *amino acid-induced germination. Further, both wild type and the *gerAA *disruption mutant germinated effectively when exposed to appropriate levels of the non-nutrient germinant Ca^2+^-DPA which by-pass the spore receptor apparatus. However, effective germination with Ca^2+^-DPA seems both strain and species specific. In order to understand and potentially control the germination behaviour of *B. licheniformis *spores, disclosure of factors involved in the transition from a dormant spore to a metabolically active proliferating cell is of prime importance. Although complete elucidation of the function and cooperation of the different germinant receptors are rather laborious, this study has taken a step in the direction of obtaining more knowledge about this less studied species. The availability of both disruption and complementation mutants will facilitate further research on the function of the GerA receptor of *B. licheniformis *MW3, as well as its potential involvement in germination triggered by alternative nutrients and cooperation with other germinant receptors. Further bioinformatic and phenotypic investigations are in progress in our laboratory and might eventually provide insight relevant for improved spore decimation techniques by the use of induced germination.

## Methods

### Bacterial strains and DNA extraction

The strains used in this study were *B. licheniformis *MW3 [50], *B. subtilis *B252 [71] and the *B. cereus *type-strain ATCC 14579 [72,73] (Table 1). *B. licheniformis *MW3 is a mutant created from *B. licheniformis *DSM13 (isogenic to ATCC 14580) with targeted deletions of the *hsdR *loci of two type I restriction modification systems making the strain readily transformable. *B. licheniformis *MW3 was used as host for creating disruption and complementation mutants of the *gerA *locus. When not stated otherwise, bacteria were cultured at 37 °C on LB agar or broth containing appropriate selective antibiotics (Table 1). Genomic DNA for PCR amplifications and sequencing was extracted from *B. licheniformis *MW3 and *B. licheniformis *NVH-1307 by a method slightly modified from [71], as follows. An overnight culture was transferred to fresh growth medium and grown at 37 °C, 225 rpm (HT-Infors AG CH-4103, Bottmingen, Switzerland), to turbidity (4-5 h). Cells from 1 ml culture was harvested by centrifugation (3 min at 16.100 × *g*), and the pellet was frozen at -20 °C. Thawed pellet was resuspended in 495 µl SET buffer (75 mM NaCl, 25 mM EDTA, 20 mM Tris, pH 7.5) and 50 µl 10 mg/ml lysozyme before incubation at 37 °C for 1 h. Further, 50 µl 10% sodium dodecyl sulfate and 5 µl 25 mg/ml proteinase K was added, and the sample was incubated at 50 °C for 2 h. At room temperature (RT), the sample was mixed with 200 µl 5 M NaCl and 700 µl of chloroform-isoamyl alcohol (24:1), and incubated with frequent inversions for 30 min. The aqueous phase was separated by centrifugation (20-30 min at 16.100-20.800 × *g*), transferred to a fresh tube, and DNA was precipitated by addition of an equal volume of isopropanol followed by centrifugation (20 min at 16.100-20.800 × *g*). The precipitate was washed with 70% ethanol and centrifuged (15 min at 16.100-16.500 × *g*), and the supernatant was removed before the precipitate was left to air dry. DNA was resuspended in 100 µl 10 mM Tris HCl buffer (pH 8.5). Plasmid DNA was purified according to the manual provided with the Plasmid Mini/Midi kits (QIAGEN^®^).

**Table 1:** Strains and plasmids used in this study

strain or plasmid	description, phenotype or genotype relevant for this study^*a*^	reference
**Strains**		
*Escherichia coli *TOP10	One Shot^® ^TOP10 electro/chemically competent *E. coli *for cloning	Invitrogen
MW3	*Bacillus licheniformis *DSM13 (Δ*hsdR1*, Δ*hsdR2*)	[50]
NVH-1307	*B. licheniformis *MW3Δ*gerAA::spc*. Sp^R^.	This study
NVH-1311	NVH-1307 with pHT315_MW3*gerA*. Sp^R ^and Em^R^.	This study
ATCC 14579	*Bacillus cereus *type strain	[72,73]
B252	*Bacillus subtilis *isolated from tap water	[71]
**Plasmids**		
pMAD	*E. coli/B. licheniformis *shuttle plasmid. Ap^R^, Em^R^, ori_*Bacillus*_^ts ^and *pclpB-bgaB*	[75]
pMAD_Sp^R^	pMAD-derivate supplemented with a Sp^R ^cassette in the *Sal*I site. Ap^R^, Em^R^, Sp^R^, ori_*Bacillus*_^ts ^and *pclpB-bgaB*	[76]
pMAD_Sp^R^Δ*gerAA*	pMAD_Sp^R^-derivate allowing substitution of parts of *gerAA *in MW3 with a Sp^R ^cassette. Ap^R^, Em^R^, Sp^R^, ori_*Bacillus*_^ts ^and *pclpB-bgaB*	This study
pHT315	*E. coli/B. licheniformis *shuttle plasmid. Ap^R ^and Em^R^	[52]
pHT315_MW3*gerA*	pHT315-derivate containing *gerA *fragment^*b *^amplified from MW3 DNA template. Ap^R ^and Em^R^	This study

*^a ^*Ap^R^; resistance to ampicillin, Em^R^; resistance to erythromycin, Sp^R^; resistance to spectinomycin, ori_*Bacillus*_^ts^; temperature-sensitive *Bacillus *origin of replication, *pclpB-bgaB*; constitutively expressed termostable β-galactosidase (allowing blue/white screening of transformants on X-Gal plates).

*^b ^gerA *fragment contains a sequence 151 bp upstream of *gerAA, gerAA, gerAB, gerAC *and 177 bp downstream of *gerAC*.

### Preparation and transformation of *B. licheniformis *electrocompetent cells

Electrocompetent *B. licheniformis *was prepared and transformed by a modified version of the protocol described by Mahillion et al.[74] as follows. A preculture in Brain Heart Infusion broth (BHI) (Oxoid, Cambridge, United Kingdom) was grown overnight at 37 °C, and 1 ml was used to inoculate 200 ml pre-warmed BHI in a 1 l Erlenmeyer. The culture was incubated 4 to 5 h at 37 °C and 150 rpm (HT-Infors AG CH-4103, Bottmingen, Switzerland) until A_600 _of 0.9-1.0 was reached (Shimadzu UV-VIS 160A, Shimadzu Europa GMBH). Cells were pelleted and washed twice with 200 ml RT autoclaved MilliQ water (MQ) by 15 min centrifugations at 3.300 and 10.400 × *g*. The pellet was resuspended in a 10 ml filter sterilised solution of freshly prepared polyethylene glycol (PEG) 6000 (Merck, Darmstadt, Germany), made by dissolving 40 g PEG6000 in 100 ml MQ. Following 15 min centrifugation at 4.080 × *g*, cells were resuspended in 0.5-1 ml of the PEG6000/MQ solution, aliquoted (100 µl) and stored at -80 °C.

Transformation was conducted by adding 2 µl plasmid to 100 µl electro competent cells thawed on ice. Following ~1 min incubation on ice, electroporation was performed at 1.4 to 2.5 kV (Eppendorf Eporator, Eppendorf AG, Hamburg, Germany or MicroPulser™, Bio-Rad, Hercules, CA), using 0.2 cm gap width electroporation cuvettes (Bio-Rad Laboratories, Hercules, CA). Before plating on selective LB-agar plates, cells were recovered in LB or S. O. C. medium (Invitrogen) at 37 °C, 150 rpm, for 4 to 5 h.

### Construction of *B. licheniformis *MW3Δ*gerAA::spc*

The shuttle vector used for construction of a spectinomycin resistant (Sp^R^) insertion deletion in the *gerAA *was pMAD_Sp^R^. This vector has been modified from pMAD [75] by insertion of a (Sp^R^)-cassette in the restriction site *Sal*I [76]. As selective antibiotics for the presence of pMAD_Sp^R ^or its derivative constructs, 100 µg/ml ampicillin and 100 µg/ml spectinomycin was used for *E. coli *TOP10 growth, and 3 µg/ml erythromycin and 250-300 µg/ml spectinomycin for *B. licheniformis *growth. This vector carries a constitutively expressed β-galactosidase gene, allowing blue-white screening on plates spread with X-Gal (40 µl 40 mg/ml 5-bromo-4-chloro-3-indolyl-β-D-galactopyranoside, VWR, BDH Prolabo). This screening was, however, not always unambiguous following long incubations of plates with *B. licheniformis *MW3 transformants, probably due to the natural precence of β-galactosidase in *B. licheniformis *DSM 13 [77]. To construct the gene replacement vector, primers (Table 2) were designed to amplify two DNA fragments, one homologous to upstream (709 bp) and one to downstream (696 bp) regions of the deletion target (567 bp) in the *gerAA*. Platinum *Taq *DNA Polymerase High Fidelity kit (Invitrogen) was used for PCR amplification with the following amplification procedure: initial denaturation for 2 min at 94°C, 30 cycles of 30 s at 94 °C, 30 s at 50 °C and 1 min at 68 °C, and final extension at 68 °C for 10 min. Primers of the upstream and downstream amplicons contained restriction sites *Bam*HI and *Eco*RI respectively (Table 2), allowing a two_step ligation into the corresponding restriction sites on either side of the (Sp^R^)-cassette in pMAD_Sp^R^. The resulting gene replacement plasmid, pMAD_Sp^R^Δ*gerAA*, was controlled for correct orientation of the upstream and downstream fragments by PCR. pMAD_Sp^R^Δ*gerAA *was introduced into *B. licheniformis *MW3 by electroporation, and allelic exchange of internal parts of *gerAA *(567 bp) with the (Sp^R^)-cassette of pMAD_Sp^R^Δ*gerAA *was allowed by double crossover. The protocol was performed as described by Arnaud et al.[75], except using growth temperatures of 37 °C following initial transformation, an incubation temperature of 45 °C and spectinomycin present during plasmid curing, and an incubation temperature of 37 °C when screening for the double crossover phenotype (spectinomycin resistant and erythromycin sensitive colonies). Chromosomal DNA was purified from a candidate colony and used in PCR amplifications (as described above) with primers hybridizing outside the cloned DNA fragment and inside the spectinomycin cassette (Table 2) to verify the deletion and insertion by sequencing. The disruption mutant was named *B. licheniformis *MW3Δ*gerAA::spc *(NVH-1307) and used in the following complementation, sporulation and germination assays.

**Table 2:** Primers used in this study

primer name	sequence^a^	Application
**Primers used in the construction/verification of the disruption mutant (NVH-1307)**		
Upper ΔgerAA F BamHI	5´- AATCGGATCCCAAGGAACACATCCATGAA-3´	Amplification of the upper fragment of pMAD_Sp^R^Δ*gerAA^b ^*
Upper ΔgerAA R BamHI	5´- TCAACAAAAATTGGGATCCGTCCATTAAA-3´	Amplification of the upper fragment of pMAD_Sp^R^Δ*gerAA^b ^*
Lower ΔgerAA F EcoRI	5´- TCTTCACCGAATTCGCTAGGCAAAGAA-3´'	Amplification of the lower fragment of pMAD_Sp^R^Δ*gerAA^b ^*
Lower ΔgerAA R EcoRI	5´- AAATGGAATTCACCGTCAAAGCTCTG-3´	Amplification of the lower fragment of pMAD_Sp^R^Δ*gerAA^b ^*
Upper ΔgerAA F2	5´- TGAAAATTTCGCCAAACACT-3´	Verification/sequencing of NVH-1307 *^b ^*
specR R	5´- TGATATGATCTTTCATTTCCATAAAAC-3´	Verification/sequencing of NVH-1307 *^c ^*
Lower ΔgerAA R2	5´- TTCGGCAGAAACATCATCAG-3´	Verification/sequencing of NVH-1307 *^b ^*
specR F	5´- ATTGAATGGACTAATGAAAATGTAAA-3´	Verification/sequencing of NVH-1307 *^c ^*
**Primers used in the construction/verification of the complementation plasmid (pHT315gerA_MW3)**		
pHT315gerAwt_SalI F1	5´-CAATCTGTCGACGTTTCCCCGTAAGCCTGATT-3´	Amplification of the *gerA *fragment^*b,d *^
pHT315gerAwt_XbaI R1	5´-GTGAGGTCTAGACCGATCGTGAAGAAAAGCAT-3´	Amplification of the *gerA *fragment^*b,d *^
ASF	5´-AAAGAAGCCTTGGAGAAGTGA-3´	Verification/sequencing of pHT315gerA_MW3*^b ^*
AOR	5´-CGCTTTGCCCTGGATATAGA-3´	Verification/sequencing of pHT315gerA_MW3*^b ^*
4AF	5´-CAATCCGCTAGGCCAGAC-3´	Verification/sequencing of pHT315gerA_MW3*^b ^*
6AF	5´-GCGGACTGAGCCTGAATATG-3´	Verification/sequencing of pHT315gerA_MW3 *^b ^*
8AF	5´-CGCTCAGGATCCGTCTAAAG-3´	Verification/sequencing of pHT315gerA_MW3*^b ^*
A9F 2/8/15	5´-CAGATCGAAGCGCTGAATTT-3´	Verification/sequencing of pHT315gerA_MW3 *^b ^*
pHT315R	5´-GGAGAAAATACCGCATCAGG-3´	Verification/sequencing of pHT315gerA_MW3*^e ^*

*^a^*The restriction sites are underlined and may differ in sequence from the template genome of Veith et. al.[48].

*^b^*The primer is complement to the genome of Veith et. al.[48].

*^c^*The primer is complement to the Sp^R ^cassette of pMAD_Sp^R^.

*^d^*The *gerA *fragment amplified contains a sequence 151 bp upstream of *gerAA, gerAA, gerAB, gerAC *and 177 bp downstream of *gerAC *relative to the genome of Veith et. al.[48].

*^e^*The primer is complement to the pHT315 vector.

**Table 3:** Ca^2+^-DPA germination in selected *Bacillus *spp

Concentration (mM)		Germination^a^			
**DPA**	**CaCl_2_**	***B. licheniformis *MW3**	***B. licheniformis *NVH-1307**	***B. subtilis *B252**	***B. cereus *ATCC 14579**

80	100	~70%	~70%	> 99%	NT^b^

45	50	~10-20%	~10-20%	~50%	> 99%

20	30	< 5%	< 5%	< 5%	~50%

^a^described as an approximate percentage of phase dark spores after screening of microscopic slides by phase contrast microscopy (100x) after 3 hours exposure in room temperature. Performed in duplicate on two individual spore batches and repeated at least twice.

^b^NT; Not Tested

### Construction of *gerA *complementation mutant

The shuttle vector used as base for trans complementation of Δ*gerAA::spc *was pHT315 [52]. As selective antibiotics for presence of pHT315 constructs, 100 µg/ml ampicillin was used for *E. coli *TOP10 growth, while 1 µg/ml erythromycin for *B. licheniformis *growth. Spectinomycin (250 µg/ml) was also supplemented for the chromosomal antibiotic resistance of NVH-1307 mutants carrying pHT315 derivatives (Table 1). To construct pHT315 complementation plasmids that harbour the *gerA *operon, DNA isolated from *B. licheniformis *MW3 was used. Primers, with *Sal*I and *Xba*I restriction sites (Table 2) were designed to amplify a 3982 bp fragment spanning from 151 bp upstream to 177 bp downstream *gerA (gerAA, gerAB, gerAC*). To ensure high fidelity, PCR amplification was performed with Phusion Hot Start II High-Fidelity DNA Polymerase kit (Finnzymes). The amplification protocol was as follows; initial denaturation for 30s at 98°C, 30 cycles of 10 s at 98 °C, 30 s at 58 °C and 2 min at 72 °C, and final extension at 72 °C for 10 min. The amplified fragments were cloned into the *Sal*I/*Xba*I restriction site of pHT315, giving the complementation plasmid pHT315_MW3*gerA*. The purified plasmid was controlled by sequencing using primers hybridizing to pHT315 and internal *gerA*. The verified plasmid was introduced into the disruption mutant (NVH-1307) by electroporation as described earlier, giving the strain *B. licheniformis *MW3Δ*gerAA::spc*pHT315_MW3*gerA *(NVH-1311). The strain was used in sporulation and germination assays.

### Sporulation

Sporulation was performed by a modified version of the sporulation protocol and medium described by van der Voort [42] as outlined below. Bacteria were pre-cultivated for 5 to 6 h in 50 ml LB-Broth with agitation (225 rpm) at 50 °C. Pre-culture of NVH-1307 was supplemented with 250 µg/ml spectinomycin, while the culture of NVH-1311 was supplemented with 250 µg/ml spectinomycin and 1 µg/ml erythromycin. Twenty µl of pre-culture was added to 100 ml sporulation medium, containing 8 g of nutrient broth (Difco, Becton, Dickinson and Company, NJ, USA) per liter, 1 μM FeSO_4_·7H_2_O (Merck KGaA, Darmstadt, Germany), 2.5 μM CuCl_2_·2H_2_O (Sigma-Aldrich, Steinheim, Germany), 12.5 μM ZnCl_2 _(Sigma-Aldrich, Steinheim, Germany), 66 μM MnSO_4_·4H_2_O (BDH Prolabo, VWR International AS, Oslo, Norway), 1 mM MgCl_2_·6H_2_O (J. T. Baker Chemicals B. V., Deventer, Holland), 5 mM (NH_4_)_2_SO_4 _(Merck KGaA, Darmstadt, Germany), 2.5 µM Na_2_MoO_4_·2H_2_O (Riedel-de Häen, Sigma-Aldrich, Seelze, Germany), 2.5 µM CoCl_2_·6H_2_O (Sigma-Aldrich, Steinheim, Germany) and 1 mM Ca(NO_3_)_2_·4H_2_O (Merck KGaA, Darmstadt, Germany). Filter sterilised Ca(NO_3_)_2_·4H_2_O, MnSO_4_·4H_2_O and FeSO_4_·7H_2_O were added to the medium after it had been autoclaved. pH was adjusted to 7.6 before autoclaving, and the pH of the final sporulation medium was 7.2. Sporulation medium of NVH-1311 was supplemented with 1 µg/ml erythromycin. The cultures were incubated with agitation (225 rpm) at 50 °C for 1 to 2 days for *B. licheniformis *strains MW3, NVH-1307 and NVH-1311, or for 2 days at 30 °C for *B. subtilis *B252 and *B. cereus *ATCC 14579 until ≥90% phase bright spores as judged by phase contrast microscopy. Spores were harvested by centrifugation for 10 min at 6000 × *g *at 4 °C, and resuspended in 10 ml cold autoclaved MQ. Washing of spores was done by centrifugation and resuspension in MQ a total of ten times. The resulting spore crops, < 10% germinated spores, were stored refrigerated in MQ. When used in the following germination studies, spore crops were between 2 and 7 months old.

### Germination assays

The spore suspension was routinely washed by centrifugation at 16.100 × *g *for 3 min prior to each experiment [78]. Spores were heat activated in MQ at 65 °C for 20 min, chilled on ice, centrifuged (16.100 × *g *for 3 min) and resuspended in 2 × germination buffer (100 mM K-phosphate buffer pH 7.2) for L-alanine germination or 1 × germination buffer (50 mM Tris HCl pH 7.4 10 mM KCl) for germination with casein hydrolysate (Merck, Darmstadt, Germany). Casein hydrolysate consists of a mixture of different amino acids (Merck Microbiology Manual 12^th ^Edition: typical amino acid content (% w/w); alanine (2.00), arginine (2.20), aspartic acid (4.40), glutamic acid (12.50), glycine (1.20), histidine (1.80), isoleucine (2.40), leucine (3.40), lysine (5.60), methionine (1.20), phenylalanine (2.50), proline (6.10), serine (2.70), threonine (2.20), tyrosine (0.60), valine (3.90)) made from acid hydrolyzation of the milk protein casein. Germination was followed as described by Hornstra et al.[13] by monitoring the reduction in absorbance at A_600 _as spores turn from phase-bright to phase dark at 30 °C in a 96-well microplate in a plate reader (Tecan Intinite M200, Grödig, Austria). The spore suspension was adjusted to an initial A_600 _of ~2 (Shimadzu UV-VIS 160A, Shimadzu Europa GMBH) prior to addition of germinant. Germinant (filter sterilised L-alanine dissolved in MQ or casein hydrolysate dissolved in 50 mM Tris HCl pH 7.4 10 mM KCl) or negative control (MQ for L-alanine germination and 50 mM Tris HCl pH 7.4 10 mM KCl for casein hydrolysate germination) was automatically injected, and the plate was shaken for 10 s prior to the first reading. A_600 _was recorded every 30 s for 142 to 170 min, with 10 s shaking in-between each measurement. The final concentration of germination buffer was 50 mM phosphatebuffer pH 7.2 or 50 mM Tris HCl pH 7.4 10 mM KCl, and final concentration of germinant was 100 mM L-alanine or 1% (w/v) casein hydrolysate. The final concentration of spores gave an initial A_600 _of ~0.7-0.8. To inhibit germination with L-alanine and potential other amino acids in the casein hydrolysate germination assay, 0.2% D-alanine (w/v, final concentration) was in some experiments added to each test well. The germination progress was described as the percentage of the initial A_600 _(% A_600i_) for each measurement point [13]. All experiments were performed in duplicates on two individual spore batches and repeated at least twice. Germination was routinely controlled by phase-contrast microscopy (Olympus BX51, Hamburg, Germany) [13].

Spore germination in Ca^2+^-DPA was performed as follows; spores were washed in cold autoclaved MQ and resuspended in germination buffer (125-250 mM Tris base, 25-100 mM DPA (2,6-Pyridinedicarboxylic acid 99%, Sigma-Aldrich, Steinheim, Germany) pH ~8) [79]. Germination was initiated by addition of excess CaCl_2_·2H_2_O (Riedel de Häen AG, Seelze, Germany), followed by incubation for 3 h with shaking at room temperature (~20°C). The final concentrations of Ca^2+^-DPA in the assay were 20-80 mM DPA 30-100 mM CaCl_2_, and the final concentration of spores gave an initial A_600 _of ~0.6-0.8. Germination was described as an approximate percentage of phase dark spores after screening of microscopic slides by phase contrast microscopy (100 x). Experiments were performed in duplicate on two individual spore batches and repeated at least twice.

### DNA sequencing and bioinformatics

DNA sequencing was performed by GATC Biotech (Konstanz, Germany) or Source BioScience (Nottingham, United Kingdom). The genomic sequence of *B. licheniformis *DSM13 [48] was accessed at http://www.ncbi.nml.nih.gov [GenBank: AE017333].

## Authors' contributions

ISL assisted in experimental design, carried out the experiments, analysed data and drafted the manuscript. CF assisted in experimental design, carried out the experiments, analysed data and assisted in drafting the manuscript. EHM assisted in drafting the manuscript. EK and KCSR carried out experiments. JTR assisted in drafting the manuscript. PEG assisted in experimental design and drafting of the manuscript. All authors read and approved the final manuscript.
